# Bicycle impalement: A case series of penetrating handlebar injuries

**DOI:** 10.1016/j.tcr.2025.101163

**Published:** 2025-04-16

**Authors:** Zoe Flyer, John Schomberg, Andreina Giron, Laura F. Goodman

**Affiliations:** a Division of Pediatric Surgery, Children's Hospital of Orange County, Orange, CA, United States; b CHOC Research Institute, Children's Hospital of Orange County, Orange, CA, United States; c Department of Surgery, University of California Irvine, Irvine, CA, United States

**Keywords:** Pediatric trauma, Penetrating trauma, Penetrating handlebars

## Abstract

**Introduction:**

Bicycles are among the most common means of transportation and recreation in children, and significant contributor to trauma in the pediatric population. Handlebars remain the most common bicycle part responsible for injuries, though most are blunt trauma. Few studies exist about bicycle impalement, or penetrating handlebar injuries, in children. The objective of this study is an overview of the evaluation, treatment and associated outcomes of these rare injuries.

**Case presentations:**

We review a total of 8 penetrating handlebar trauma cases in children at our pediatric trauma center over the course of 4 years. In the same time frame, our pediatric trauma center treated 2506 patients for bicycle-related trauma. Institutional review board (IRB) approval was obtained per protocol prior to the study. All 8 penetrating handlebar cases were secondary to impalement with the hand-brake portion of bicycle handlebars. Age, gender, immunization status, imaging, type of procedural intervention, medications given and outcomes at follow up visits are reviewed.

**Discussion:**

All 8 cases reviewed were in male patients, median age 9. 6 patients presented with penetrating injuries to the lower extremity, and 2 patients with injuries to the abdomen. All 8 cases were up to date on immunizations and received a dose of intravenous antibiotics at presentation. Plain film radiographs and CT images were obtained based on patient's clinical findings, and type of procedural intervention pursued was ultimately provider-dependent. All 8 patients were discharged on oral antibiotics, and all were noted to be healing well without signs of infection at follow-up.

**Conclusion:**

While rare, prompt recognition, evaluation and treatment of penetrating handlebar injuries in the pediatric population is imperative to improving clinical outcomes. Immediate evaluation includes patient's immunization status, decision to obtain imaging and operative intervention when indicated. Post-operative prophylactic antibiotics are not mandatory in cases of complete foreign body removal. Review of a larger number of penetrating handlebar cases could be pursued to further delineate best-practice protocols for this population.

## Introduction

Bicycles are a common means of transportation, exercise and recreation among children. Bicycle crashes, however, are a significant contributor to trauma and associated morbidity in the pediatric population [[1], [2], [3]]. McAdams et al. reported that as many as 608 children with bicycle-related injuries present to US emergency departments every day [4,5]. Another national study reported that bicycles are second-most common cause for transportation-related injuries in children, after motor vehicles [6]. Bicycle trauma is generally categorized into two different injury mechanisms: falling from the bicycle or direct impact with the bicycle [7]. In instances where patients make direct impact with the bicycle, the part of the bicycle responsible is most commonly the handlebars [7]. Studies reveal that handlebar injuries often affect the abdomen, and diagnosis and treatment of associated intraabdominal injuries can be delayed in the setting of few specific radiographic or clinical findings [8,9]. The lack of specific radiographic or clinical findings has ultimately led to the severity of handlebar injuries being frequently underestimated [2,8].

Much of the published literature depicts handlebar injuries in children who have experienced blunt trauma [2,[10], [11], [12]], and the most common intraabdominal injuries are to the liver, spleen and pancreas [13]. Few studies report the unique presentation of penetrating handlebar injuries in the pediatric population (Table 1) [11,[13], [14], [15]]. In an effort to augment the rare reports of penetrating trauma in the established literature, this paper provides a case series of 8 children presenting to a pediatric trauma center with penetrating handlebar injuries over the course of 4 years (Table 2). Institutional review board (IRB) approval was obtained per protocol prior to the study.

**Table 1:** Existing case reports of penetrating handlebar injuries in children.

	Number of subjects	Gender (M/F)	Average age	Location of injuries	Median length of hospital stay (days)	Post-operative antibiotics	Wound complications
Thompson et al	1	M	14	Groin, penis and scrotum	2	Yes	None
Ramos-Izarra et al	2	M	10.5	Groin, thigh	18	Yes	None
Debbink et al	1	F	13	Abdomen, ureter	5	Yes	None
Krishan et al	1	F	8	Abdomen, iliac wing	3	Yes	None

**Table 2:** Characteristics of This Series' Pediatric Patients with Penetrating Handlebar Injuries.

	Male (*n* = 8)	Female (*n* = 0)
Median age (years)	9 (8–15)	n/a
Helmet use	2	n /a
Immunizations up to date	7	n/a
Foreign body present on arrival	3	n/a
Imaging obtained	7	n/a
Penrose drain utilized	3	n/a
Median length of stay (days)	2 (1–4)	n/a
Wound complications	0	n/a

## Case presentations

### Case 1

An otherwise healthy 10-year-old boy presented to the emergency department via ambulance after a fall from his bicycle. The patient reported falling off while riding his bike, directly striking his body on the handbrake lever on his handlebars. The handbrake subsequently penetrated the right lower quadrant of the patient's abdomen. Emergency medical services (EMS) dismantled the handlebars from the bike and left the impaled object in place during transport. Whether or not patient had been wearing a helmet is unknown, and immunizations were up to date.

**Fig. 1:**
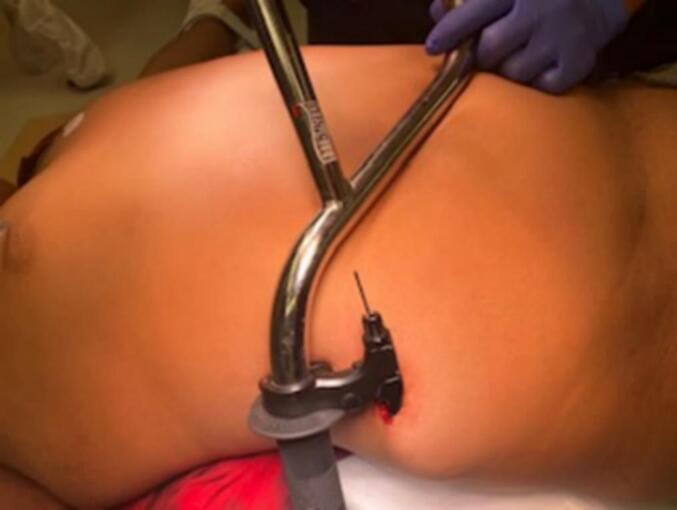
Case 1, handbrake penetrating right lower quadrant.

**Fig. 2:**
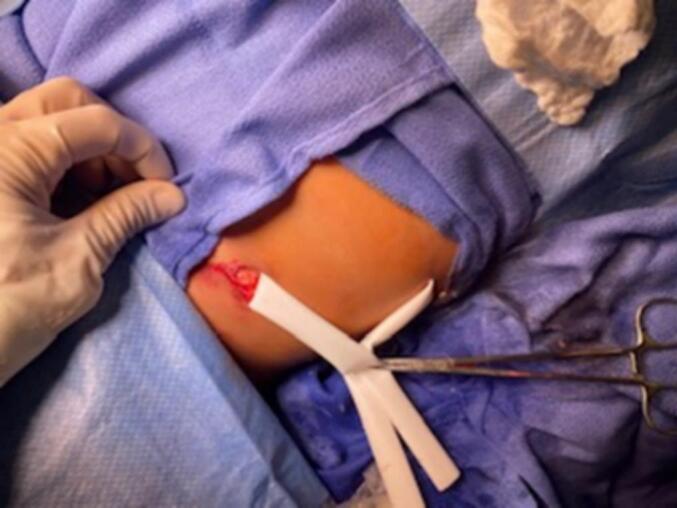
Case 1, right lower quadrant wound after irrigation and penrose drain placement.

On initial examination, the handbrake remained in place to the right lower quadrant and was noted to be hemostatic (Fig. 1). Laboratory tests that were drawn and vital signs upon arrival were all noted to be unremarkable and within normal limits. The patient received one dose of intravenous cefazolin and was subsequently taken directly to the operating room for removal of the handbrake, which was noted to be lodged in subcutaneous fat without violation of the muscle fascia or vasculature. The wound was irrigated and left open with a 1-in. penrose drain (Fig. 2). Patient was admitted for observation and discharged on post-operative day 1 with instructions to complete a 7-day course of cephalexin. He was seen in clinic on post-operative day 6 for wound check and penrose removal, and complete wound healing without complication was noted on post-operative day 20.

### Case 2

A 9-year-old boy with no significant medical history presented to the emergency department after a fall from his bicycle, that resulted in the hand brake of his handlebars penetrating his left thigh. The patient was not wearing a helmet during the incident, and immunization status was unknown. Tdap was subsequently administered on arrival.

On exam, patient presented with a 6 cm wound to the left thigh, just inferior to the left inguinal crease. The wound was noted to be hemostatic, and dorsalis pedis and posterior tibial pulses were palpable on the affected side. Plain film radiograph of the pelvis ruled out bony injury, and CT angiogram of the left lower extremity was obtained to ensure no direct injury to the vasculature.

After one dose of intravenous cefazolin, patient was taken to the operating room for exploration and washout of the wound that was noted to track 15 cm underneath the skin along the subcutaneous plane. Muscles were intact and no injury to the groin vessels was noted. The wound was closed with multiple layers of absorbable sutures, before a ¼-inch penrose drain was applied underneath the skin, closed with absorbable monofilament. The patient was admitted for observation and discharged on post-operative day 3 with instructions to finish a 7-day course of oral cephalexin. The patient was seen in clinic 2 weeks later at which time the penrose drain was removed, and the wound was noted to be well approximated and well healed without evidence of infection.

### Case 3

**Fig. 3:**
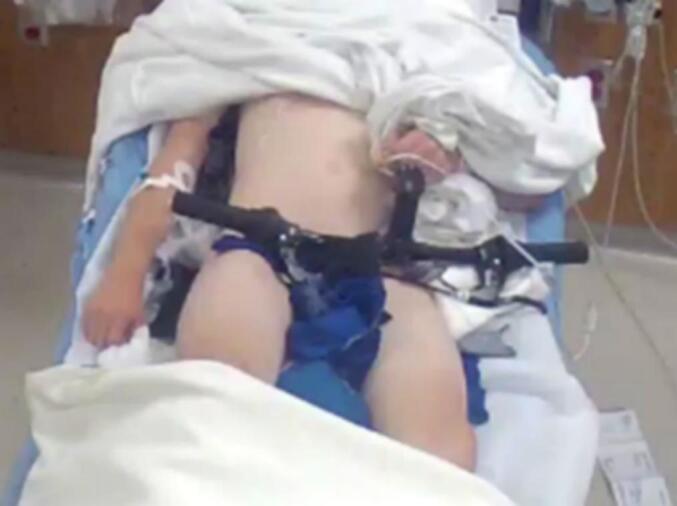
Case 3, handbrake penetrating right anterior thigh.

A 12-year-old boy presented to the emergency department via ambulance after a fall from his bicycle. The patient had been undergoing concurrent outpatient workup for a possible platelet dysfunction, after abnormal findings on a platelet function assay (PFA). The patient attempted to go off a jump, causing him to lose control of his handlebars which twisted, and the patient landed atop the hand brake. The hand brake punctured the patient's right thigh. The handlebars were dismantled from bicycle to leave the impaled foreign body in place during transport the emergency department (Fig. 3). Helmet status was unknown, and immunizations up to date.

Laboratory tests that were drawn and vital signs obtained upon patient arrival were largely within normal limits. In the setting of patient's known platelet dysfunction, thromboelastography was obtained, and the patient received 1 unit of platelets. A plain film radiograph was obtained of the right lower extremity and pelvis, and was normal. Orthopedic surgery was consulted due to a high suspicion for open fracture, despite no bony injury noted on imaging. Vascular surgery was notified prior to operative management should vascular injury be encountered during the procedure.

The patient received one dose of intravenous cefazolin and was subsequently taken to the operating room for removal of the hand brake and washout. Hemostasis was achieved with gel-foam thrombin, and the wound was closed with multiple layers of absorbable suture. He was admitted overnight for observation and discharged home on post-operative day 1 with instructions to complete a 7-day course of cephalexin. The patient was evaluated on post-operative day 7 in clinic, noted to be healing well with signs of improving, mild ecchymosis.

### Case 4

An otherwise healthy 15-year-old boy presented to the emergency department with EMS after a fall from his bicycle. Patient had been riding his mountain bike on a trail when he lost control of his handlebars before they twisted and the hand brake impaled his left groin. The patient was wearing a helmet at the time of the incident, and immunizations were up to date on arrival.

Upon presentation, the patient was noted to have a 4 cm laceration in the suprapubic region just above the left groin, with a slow ooze of blood. Laboratory tests drawn and vitals measured upon arrival were unremarkable and within normal limits. Dorsalis pedis and posterior tibial pulses were palpable on the affected side and symmetric. Plain film radiographs of the pelvis were obtained and revealed no evidence of bony injury. CT abdomen/pelvis angiogram was obtained to rule out direct injury to the femoral and iliac vessels, as small area of adjacent active hemorrhage was noted. The patient was given a dose of intravenous cefazolin and taken to the operating room for wound exploration, washout and repair. Plastic surgery was consulted in the event that a muscle flap would be needed to cover the femoral vessels, though no flap was needed. The injury was noted to track 8 cm medially through the subcutaneous tissues, just below the inguinal ligament. There were superficial tears to the pectineus and adductor longus muscles. Small branches exposed off of the greater saphenous vein were suture ligated. The wound was closed with multiple layers of absorbable suture before a ¼-inch penrose drain was placed below the superficial subcutaneous layer, and skin was reapproximated with absorbable suture. Bacitracin was applied.

The patient was admitted for observation and discharged on post-operative day 2 after the drain was removed. The patient finished a 7-day course of oral cephalexin and was seen in clinic 3 weeks later. The wound was well approximated and well healed without signs of infection, distal vascular exam was unchanged.

### Case 5

**Fig. 4:**
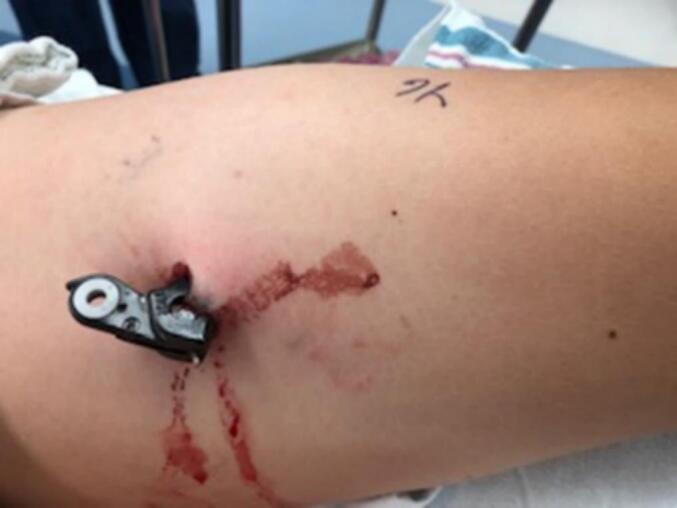
Case 5, handbrake penetrating left lateral thigh.

A 12-year-old boy with no significant medical history presented to the emergency department via ambulance after a bicycle collision. The patient had been riding his bicycle with friends when they crashed into one another, and the hand brake from an opposing handlebar punctured his left thigh. The hand brake was separated from the rest of the handlebars and transported in situ (Fig. 4). The patient's helmet status was unknown and immunizations were up to date.

Labs drawn and vitals taken upon admission were unremarkable and within normal limits. A CT angiogram of the left lower extremity was obtained to rule out vascular injury, and was normal. The patient was subsequently taken to the operating room after one dose of intravenous cefazolin for removal of the foreign body and washout. The wound was noted to be 3 cm × 3 cm, extending into subcutaneous fat, and degloving laterally along the left lower extremity for approximately 10 cm. The wound was copiously irrigated and loosely closed in layers using absorbable and permanent sutures over a ¼-inch penrose drain.

The patient was admitted for observation and discharged on post-operative day 1 with instructions to complete a 7-day course of cephalexin. He was seen in clinic on post-operative day 6, at which point nylon sutures and the penrose drain were removed to good effect.

### Case 6

An otherwise healthy 9-year-old boy presented to the emergency department with family after a fall from his bicycle. The patient had been riding his bike on his residential street when he fell and sustained a laceration to his right thigh secondary to impalement with the hand brake of his handlebars, which was removed in the field. The patient was not wearing a helmet and was noted to be up to date on vaccinations. The patient ambulated on scene without difficulty.

On arrival, laboratory tests drawn and vitals collected were noted to be within normal limits. There was no clinical evidence of vascular or neurological abnormalities. The wound was noted to be hemostatic with evidence of exposed subcutaneous fat. A plain film radiograph of the right lower extremity was obtained, which revealed subcutaneous emphysema consistent with soft tissue injury and no evidence of bony injury.

The wound was noted to be 6 cm × 2.5 cm × 3.5 cm, deep muscle fascia intact, and copiously irrigated after one dose of intravenous cefazolin. The wound was reapproximated with multiple layers of absorbable suture. Patient was discharged home with instructions to complete a 7-day course of cephalexin. He was seen in clinic 14 days after initial presentation for removal of sutures, at which point the wound edges were noted to be well approximated and well healed.

### Case 7

An otherwise healthy 8-year-old boy was transferred to the emergency department from an outside hospital after a fall from his bicycle. The patient had reportedly been riding his bike when he fell from his seat, landing directly on the hand brake of his handlebars. The hand brake penetrated the patient's right upper quadrant and was removed by EMS in the field upon arrival. Patient was noted to be helmeted and immunizations up to date.

**Fig. 5:**
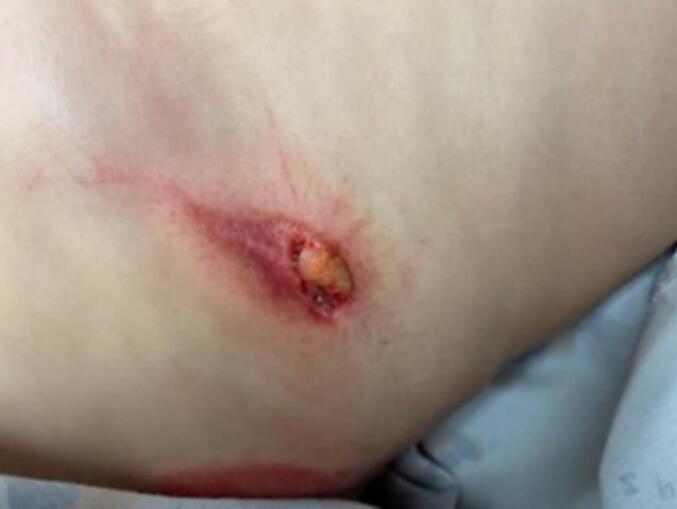
Case 7, right upper quadrant wound with protruding subcutaneous fat.

Upon arrival, laboratory tests drawn and vital signs collected were unremarkable and within normal limits. The wound at the right upper quadrant was noted to be approximately 1 cm in size, and was hemostatic with a small amount of protruding subcutaneous fat (Fig. 5). A CT abdomen with IV contrast was obtained to ensure no penetration of the abdominal fascia, and a small amount of subcutaneous emphysema was seen consistent with superficial injury.

After administration of intravenous cefazolin, the wound was washed out at the bedside and left open, with a petroleum dressing applied. The hospital's wound care team was consulted, and patient was discharged on hospital day 2 with instructions to change an absorbent foam dressing four times daily for 7 days and complete a 7 day course of cephalexin. Patient was seen in clinic 6 days after initial presentation, and wound was noted to be healing well without signs of infection.

### Case 8

A 9-year-old boy with no significant past medical history presented via ambulance after an electric bicycle crash at the park. The patient had been riding his bike at approximately 5-10mph when he hit a pothole, causing him to lurch forward over his handlebars and the hand brake to impale his left thigh. The patient was not wearing a helmet, and was up to date on immunizations.

A bystander had removed the hand brake from patient's left thigh prior to transport with EMS. The patient's left thigh wound was hemostatic on arrival. Laboratory tests and vital signs were unremarkable and within normal limits. A plain film radiograph on the left lower extremity was obtained and revealed a small amount of subcutaneous emphysema consistent with soft tissue injury. No bony abnormalities noted.

After one dose of intravenous cefazolin, the patient's wound, noted to be 3 cm in depth, was irrigated at the bedside and closed with permanent simple interrupted sutures. Bacitracin was applied, and patient was discharged with instructions to complete a 10-day course of cephalexin. The patient was seen in clinic 10 days later for suture removal, at which point wound was noted to be well approximated and well healed.

## Discussion

Penetrating handlebar injuries are a rare but potentially serious manifestation of pediatric bicycle trauma. Out of the 2506 bicycle-related injuries treated at our pediatric trauma center over the course of 4 years, 8 cases involved penetrating handlebar trauma. Previous studies reveal that the majority of handlebar injuries result from direct impact with handlebars, or when the bicycler is ejected over the handlebars [6,10,17]. However, most cases in the literature are blunt injuries and not impalements. The 8 cases examined in our study were impalements, and all 8 of these penetrating injuries involved the hand brake portion of the handlebars. It has been surmised that the severity of handlebar injuries is often related to the handlebar's small cross-sectional area, leading to a forceful transfer to energy even when injuries occur at low-end speed [2]. This theory would suggest that mechanical risk of impalement is likely heightened with the hand brake portion of a handlebar, as the cross-sectional area is even more narrow, acting as a stronger focal point and threat to penetration. The case series substantiates this prediction, as all 8 bicycle-related handlebar injuries examined involved penetrating trauma due to the hand brake.

Notably, all 8 of the penetrating handlebar trauma cases reported in this pediatric series were male subjects. These findings are consistent with established literature that reveals that the majority of handlebar trauma is seen in male patients [2,10,17,18]. Some studies attributed increased bicycle trauma in male patients due to males' increased propensity to take risks, reduced perception of risk and restraint [[19], [20], [21]]. The delay in diagnosis and treatment that has previously been reported in blunt handlebar injuries [8,9] has been ameliorated in recent studies that report the need for heightened suspicion of intra-abdominal injury should a handlebar imprint be observed on the patient [22]. This approach to patient evaluation was less applicable in this case series, as we report on penetrating injuries where 3 of the 8 cases presented with foreign body still in place. Five of the 8 cases presented after the embedded object had been removed in the field, despite the recommendation that impaled foreign bodies be left in-situ as they could be preventing hemorrhage by acting to tamponade nearby vasculature [23,24].

While no single published protocol is applicable to triaging all penetrating trauma, it is generally recommended that patients who present with penetrating injuries are up to date with the TDap immunization schedule for tetanus prophylaxis [6,25]. 8 of the patients presented in this series were ensured to have immunizations up to date on arrival. 1 patient's immunization status was unknown, and Tdap was administered promptly at presentation. A study produced by Waller et al. reports that imaging is typically not required if the foreign body is visible on arrival [6]. Plain film radiographs are generally the most readily available and economically sound methods for evaluation foreign bodies that are radiopaque [6,16]. The decision to obtain imaging for the injuries in this case series was based on individual providers' perceived severity and location of injury. CT angiogram was obtained in the 3 cases where patients presented with handbrake injuries to the groin and thigh, questionably near the femoral bundle and iliac vessels. CT abdomen with IV contrast was obtained in the instance where the patient presented with an injury to the right upper quadrant, to ensure no violation of the abdominal fascia that would encourage the provider to perform laparoscopy or laparotomy. Bony injury was ruled out in extremity cases with plain film radiographs.

Infection is the most common post-operative complication after foreign body removal. Signs of active infection, such as cellulitis or abscess, is an absolute contraindication to wound closure [26,27]. Wounds secondary to foreign body penetration can be closed so long as the wound cavity is copiously and completely irrigated [28,29]. Whether or not to close a wound after clean foreign body removal is subject to the provider's clinical judgement. Studies suggest that closure of a wound over a penrose drain after adequate washout does not reduce the incidence of surgical site infections [[30], [31], [32]]. The decision to close wounds over a penrose drain, in 4 instances of this series, was provider dependent, likely related to the extent of deep injury and concerns that the wound could not be irrigated in its entirety. Furthermore, post-operative prophylactic antibiotics are not recommended when the penetrating object has been removed in its entirety [25,33]. Despite this recommendation, all 8 cases of this series were discharged on a post-operative course of oral antibiotics. All 8 patients were seen post-operatively in clinic and noted to have healing wounds without signs of infection or wound complications.

## Conclusion

While uncommon, penetrating handlebar injuries are a serious manifestation of bicycle trauma in the pediatric population. Prompt recognition, evaluation and treatment of penetrating handlebar injuries is imperative to improving clinical outcomes; including patient's immunization status, decision to obtain imaging and operative intervention when indicated. Post-operative prophylactic antibiotics are not mandatory in cases of complete foreign body removal. Review of a larger number of penetrating handlebar cases could be pursued to further delineate best-practice protocols for this population.

## CRediT authorship contribution statement

**Zoe Flyer:** Writing – original draft, Conceptualization. **John Schomberg:** Formal analysis. **Andreina Giron:** Writing – review & editing. **Laura F. Goodman:** Writing – review & editing, Conceptualization.

## Financial support statement

This research did not receive any specific grant from funding agencies in the public, commercial or not-for-profit sectors.

## Declaration of competing interest

The authors declare that they have no known competing financial interests or personal relationships that could have appeared to influence the work reported in this paper.
